# COVID-19 no Período Pós-Operatório Inicial de Transplante Cardíaco - Experiência Inicial

**DOI:** 10.36660/abc.20200868

**Published:** 2021-01-27

**Authors:** Gustavo Pampolha Guerreiro, Lucas Molinari Veloso da Silveira, Valdano Manuel, Samuel Padovani Steffen, Fernando Bacal, Fabio Antonio Gaiotto, Fabio Biscegli Jatene

**Affiliations:** 1 Hospital das Clínicas Faculdade de Medicina Universidade de São Paulo São PauloSP Brasil Instituto do Coração do Hospital das Clínicas da Faculdade de Medicina da Universidade de São Paulo (Incor-HCFMUSP), São Paulo, SP – Brasil

**Keywords:** Covid-19/complicações, Pandemia, Fatores de Risco, Síndrome Respiratória Aguda, Transplante de Coração, Imunossupressão

## Introdução

A pandemia da doença de coronavírus 2019 (COVID-19) está aumentando rapidamente em todo o mundo. O Brasil é o segundo país com mais casos, sendo considerado o epicentro da América do Sul.^1^

A doença cardiovascular é conhecida por ser um importante fator de risco para susceptibilidade à infecção, gravidade da doença e mau prognóstico em COVID-19. Os receptores de transplante cardíaco (TC) podem ter um risco aumentado devido às suas comorbidades; no entanto, foi teorizado que a imunossupressão pode protegê-los da tempestade de citocinas que é responsável por desfechos piores.^2 , 3^ Por outro lado, a infecção pelo coronavírus da síndrome respiratória aguda grave 2 (SARS-CoV-2) tem sido relatada em pacientes com TC com apresentação da doença semelhante à população geral, questionando o teorizado mecanismo de proteção da imunossupressão.^4 - 6^

Apresentamos aqui quatro casos de COVID-19 durante o período pós-operatório (PO) inicial de TC, com diferentes desfechos em curto prazo, incluindo um óbito devido a complicações respiratórias.

## Relatos de Caso

### Caso 1

**Figura 1:**
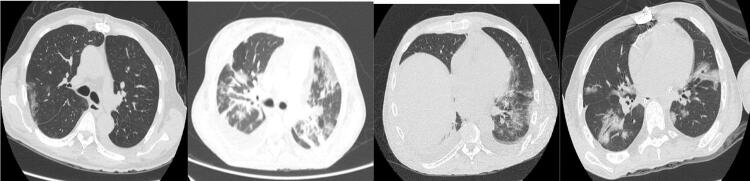
– Tomografia computadorizada de tórax revelando opacidades em vidro fosco em caso 1 (A), caso 2 (B), caso 3 (C) e caso 4 (D).

Um paciente de sexo masculino de 51 anos de idade, no 50º dia PO, apresentou um quadro de dor torácica com características pleuríticas. A tomografia computadorizada de tórax revelou padrão de vidro fosco ( Figura 1A ), com diagnóstico de COVID-19 ( Tabela 1 ). Nenhum tratamento específico foi necessário. A ecocardiografia transtorácica (ETT) revelou fração de ejeção do ventrículo esquerdo (FEVE) normal (67%). O paciente recebeu alta para casa após tratamento para complicações infecciosas menores relacionadas ao estado imunossupressor.

**Tabela 1:** – Características de linha de base e testes laboratórios no momento do diagnóstico de COVID-19

	**Paciente 1**	**Paciente 2**	**Paciente 3**	**Paciente 4**
**Idade (anos)**	55	22	48	31
**Sexo (masculino/feminino)**	Masculino	Feminino	Masculino	Masculino
**Etiologia da doença cardíaca**	Chagásica	Dilatada	Chagásica	CAVD
**INTERMACS**	1	2	2	3
**Condição pré-operatório**	Inotrópico + ECMO	Inotrópico + BIA	Inotrópico + BIA	Inotrópico
**Imunossupressão durante COVID-19**	Corticosteroides + Micofenolato + Ciclosporina	Corticosteroides + Ciclosporina	Corticosteroides	Corticosteroides + Micofenolato
**TIH pré-TC (dias)**	14	80	58	143
**Tempo de isquemia fria (minutos)**	212	261	146	161
**FEVE PO de TC (%)**	67	60	63	65
**Diagnóstico PO de COVID-19 (dias)**	50	45	24	5
**Apresentação da COVID-19 **	Leve	Moderada	Moderada	Grave

*BIA: balão intra-aórtico; CAVD: cardiomiopatia arritmogênica do ventrículo direito; COVID-19: doença de coronavírus 2019; ECMO: oxigenação por membrana extracorpórea; FEVE: fração de ejeção do ventrículo esquerdo; INTERMACS: Interagency Registry for Mechanically Assisted Circulatory Support; PO: pós-operatório; TC: transplante cardíaco; TIH: tempo de internação hospitalar.*

### Caso 2

Uma paciente de sexo feminino de 22 anos de idade apresentou disfunção primária do enxerto, necessitando de oxigenação por membrana extracorpórea (ECMO) para a recuperação. Após o desmame da ECMO no 7º dia PO, a paciente apresentou febre que levou ao diagnóstico de COVID-19 ( Tabela 1 ). Necessitou de oxigenoterapia, porém sem ventilação mecânica ou tratamento específico. A tomografia computadorizada ( Figura 1B ) revelou um padrão de vidro fosco. A paciente recebeu alta após anticoagulação por leve embolia pulmonar. A última ETT mostrou FEVE 60%.

### Caso 3

Um paciente de sexo masculino de 48 anos de idade, durante internação por insuficiência cardíaca descompensada, apresentou sintomas respiratórios e tomografia computadorizada de tórax sugestivos de COVID-19; no entanto, foi excluída após 3 testes negativos. O PO inicial transcorreu sem intercorrências até o 21º dia PO ( Figura 1C ), quando apresentou febre e foi diagnosticado com COVID-19 ( Tabela 1 ). Oxigenoterapia suplementar foi necessária, mas não ventilação mecânica. O paciente recebeu azitromicina durante o seu tratamento para COVID-19. O paciente recebeu alta com FEVE normal avaliada por ETT (63%).

### Caso 4

Um paciente de sexo masculino de 31 anos de idade, no 5º dia PO, apresentou tosse e delírio. A tomografia computadorizada de tórax revelou imagens em vidro fosco em ambos os pulmões ( Figura 1C ) e o teste para COVID-19 foi positivo ( Tabela 1 ). Foi necessária oxigenoterapia suplementar e o paciente progressivamente piorou, necessitando de ventilação mecânica. O paciente recebeu azitromicina durante seu tratamento para COVID-19. A última FEVE avaliada por ETT foi normal (65%). O paciente evoluiu para óbito no 12º dia PO devido a insuficiência respiratória aguda.

## Discussão

A pandemia de infecção por SARS-CoV-2 está aumentando dramaticamente em todo o mundo.^1^ As cirurgias eletivas têm sido canceladas e os leitos de enfermaria/UTI normalmente dedicados a cuidados pré- e pós-operatórios têm sido designados para pacientes com COVID-19. Os cirurgiões cardíacos e cardiologistas estão enfrentando sérios problemas na tomada de decisões para tratar pacientes cirúrgicos neste período, uma vez que é necessário equilibrar o risco de morte cardiovascular devido à intervenção tardia, o risco de operar um paciente em incubação ou no período assintomático de infecção por COVID-19 e o risco de infecção durante a internação hospitalar após a cirurgia cardíaca.^7^

Em relação aos pacientes com insuficiência cardíaca, o desafio é ainda maior, pois, devido à descompensação cardíaca, esses pacientes frequentemente requerem longas internações, as quais aumentam o risco de COVID-19. De 2010 a 2018, 44% dos pacientes estavam internados no momento do TC.^8^ Durante a pandemia, muitos centros de TC estão reavaliando as suas listas de espera, dando prioridade aos pacientes com menor expectativa de vida ou pacientes hospitalizados que têm contra-indicação para dispositivo de assistência ventricular esquerda (DAVE) de longa duração.^9^ Infelizmente, essa estratégia não é factível para todos os centros devido à falta de recursos, especialmente durante a pandemia.

Os nossos receptores de TC incluem em sua maioria pacientes hospitalizados e priorizados, e DAVE de longa duração não foi possível. A maioria dos nossos pacientes submetidos ao TC nos últimos 10 anos foi hospitalizada no momento do TC. Apesar de todas as medidas preventivas tomadas durante a internação de acordo com os protocolos institucionais, esses pacientes apresentam alto risco de serem infectados pelo SARS-CoV-2.

De acordo com a classificação de estadiamento proposta por Siddiqi e Mehra, apenas um de nossos pacientes apresentou COVID-19 grave.^10^ Os três primeiros pacientes apresentaram formas leves e moderadas, não necessitando de tratamento específico ou intensivo. Apenas dois pacientes receberam azitromicina. O último paciente veio a óbito devido a insuficiência respiratória aguda. Com base em nossa experiência limitada e outros relatórios publicados, a COVID-19 pode ter apresentação semelhante em receptores de TC durante o período PO inicial (de forma leve a grave), em comparação tanto aos receptores de TC no período PO tardio quanto à população geral.^4 - 6^

Até onde sabemos, esta série de casos é a primeira a relatar resultados em receptores de TC que contraíram a COVID-19 durante o período PO inicial e nossa experiência mostrou apresentações da doença semelhantes em comparação aos pacientes não receptores de TC previamente relatados. Séries maiores são necessárias para entender essa hipótese melhor. Atualmente, parece que o TC deve ser considerado para aqueles pacientes que não podem receber alta para casa em centros onde o DAVE de longa duração não é disponível, considerando os riscos e benefícios individuais, avaliados para cada paciente e realidade local.
