# Supplementary material for: COVID-19 no Período Pós-Operatório Inicial de Transplante Cardíaco - Experiência Inicial
Source: Arq Bras Cardiol. 2021 Jan 27;116(1):144–6. [Article in Portuguese] doi: 10.36660/abc.20200868 (PMC8159498; doi:10.36660/abc.20200868)
Supplement: Translation

# Translation: COVID-19 in Early Postoperative Heart Transplantation - Initial Experience

**Authors:** Gustavo Pampolha Guerreiro, Lucas Molinari Veloso da Silveira, Valdano Manuel, Samuel Padovani Steffen, Fernando Bacal, Fabio Antonio Gaiotto, Fabio Biscegli Jatene

**Affiliations:** 1 Brazil Instituto do Coração do Hospital das Clínicas da Faculdade de Medicina da Universidade de São Paulo (Incor-HCFMUSP), São Paulo, SP – Brazil

**Keywords:** Covid-19/complications, Pandemics, Risk Factors, Severe Acute Respiratory Syndrome, Heart Transplantation, Imunossupression

## Introduction

The coronavirus disease (COVID-19) pandemic is rapidly increasing worldwide. Brazil is the country with the second highest number of cases, and it is considered South America’s epicenter.^1^

Cardiovascular disease is known to be an important risk factor for infection susceptibility, illness severity, and poor prognosis in COVID-19. Heart transplantation (HT) recipients may have an increased risk due to their comorbidities; however, it has been theorized that immunosuppression might protect them from the cytokine storm responsible for worse outcomes.^2 , 3^On the other hand, infection by severe acute respiratory syndrome coronavirus 2 (SARS-CoV-2) has been reported in HT patients with disease presentation similar to general population, questioning the theorized immunosuppression protective mechanism.^4 - 6^

Herein, we present four cases of COVID-19 during the early postoperative (PO) HT period, with different short-term outcomes, including one death due to respiratory complications.

## Case Reports

### Case 1

A 51-year-old male patient, on PO day 50, presented chest pain with pleuritic characteristics. His chest computed tomography (CT) showed a ground glass pattern ( Figure 1A ), and was diagnosed with COVID-19 ( Table 1 ). No specific treatment was required. Transthoracic echocardiography (TTE) showed normal (67%) left ventricle ejection fraction (LVEF). He was discharged home after receiving treatment for minor infectious complications related to immunosuppressive status.

**Figure 1:** – Chest computed tomography revealing peripheral ground glass opacities in case 1 (A), case 2 (B), case 3 (C) and case 4 (D).

**Table 1:** – Baseline characteristics and laboratory tests at the time of COVID-19 diagnosis

	**Patient 1**	**Patient 2**	**Patient 3**	**Patient 4**
**Age (years)**	55	22	48	31
**Sex (male/female)**	Male	Female	Male	Male
**Heart disease etiology**	Chagasic	Dilated	Chagasic	ARVC
**INTERMACS**	1	2	2	3
**Preoperative condition**	Inotropic + ECMO	Inotropic + IABP	Inotropic + IABP	Inotropic
**Immunosuppression during COVID-19**	Corticosteroids + Mycophenolate + Cyclosporine	Corticosteroids + Cyclosporine	Corticosteroids	Corticosteroids + Mycophenolate
**LOS pre-HT (days)**	14	80	58	143
**Cold ischemia time (minutes)**	212	261	146	161
**LVEF PO HT (%)**	67	60	63	65
**PO COVID-19 diagnosis (days)**	50	45	24	5
**COVID-19 presentation**	Mild	Moderate	Moderate	Severe

*ARVC: arrhythmogenic right ventricular cardiomyopathy; COVID-19: coronavirus disease 2019; ECMO: extracorporeal membrane oxygenation; HT: heart transplant; IABP: intra-aortic balloon pump; INTERMACS: Interagency Registry for Mechanically Assisted Circulatory Support; LOS: length of stay; LVEF: left ventricular ejection fraction; PO: postoperative.*

### Case 2

A 22-year-old female patient had primary graft dysfunction requiring extracorporeal membrane oxygenation (ECMO) for recovery. After weaning from ECMO on PO day 7, she presented fever that lead to COVID-19 diagnosis ( Table 1 ). She required oxygen therapy, without mechanical ventilation or specific treatment. CT scan ( Figure 1B ) showed a ground glass pattern. The patient was discharged after anticoagulation due to minor pulmonary embolism. Last TTE showed 60% LVEF.

### Case 3

A 48-year-old male patient, during hospitalization for decompensated heart failure, presented respiratory symptoms and chest CT suggestive of COVID-19; however, this was excluded after 3 negatives tests. Early PO was uneventful until PO day 21 ( Figure 1C ), when he presented fever and was diagnosed with COVID-19 ( Table 1 ). Supplementary oxygen therapy was required, but not mechanical ventilation. The patient received azithromycin during his COVID-19 treatment. He was discharged with normal LVEF assessed by TTE (63%).

### Case 4

A 31-year-old male patient, on PO day 5, presented cough and delirium. Chest CT showed ground glass images in both lungs ( Figure 1C ) and he tested positive for COVID-19 ( Table 1 ). Supplementary oxygen therapy was needed, and he progressively got worse, requiring mechanical ventilation. The patient received azithromycin during his COVID-19 treatment. Last LVEF assessed by TTE was normal (65%). The patient died on PO day 12 due to acute respiratory failure.

## Discussion

The pandemic of SARS-CoV-2 infection is dramatically increasing worldwide.^1^Elective surgeries have been cancelled and ward/ICU beds dedicated to pre- and postoperative care have been designated for patients with COVID-19. Cardiac surgeons and cardiologists are facing serious issues in making decisions to treat surgical patients in this period, since it is necessary to balance the risk of cardiovascular death due to delayed intervention, the risk of operating a patient in incubation or asymptomatic period of COVID-19 infection, and the risk of being infected during hospitalization after cardiac surgery.^7^

Concerning patients with heart failure, the challenge is even greater, because, due to cardiac decompensation, these patients frequently require long hospitalizations, which increase the risk of COVID-19. From 2010 to 2018, 44% of patients were hospitalized at the time of HT.^8^During the pandemic, many HT centers are reassessing their waiting lists, prioritizing patients with lower life expectancy or hospitalized patients who have contraindications for durable left ventricular assist device (LVAD).^9^Unfortunately, this strategy is not feasible for all centers due to a lack of resources, especially during the pandemic.

Our HT recipients include mostly hospitalized and prioritized patients, and durable LVAD was not possible. Most of our patients who underwent HT during the last 10 years were hospitalized at the time of HT. Despite all the preventive measures taken during hospitalization according to institutional protocols, these patients are at high risk of being infected by SARS-CoV-2.

According to the staging classification proposed by Siddiqi and Mehra, only one of our patients had severe COVID-19.^10^The first three patients presented with mild and moderate forms, not requiring specific or intensive care treatment. Only two patients received azithromycin. The last patient died due to acute respiratory failure. Based on our limited experience and other published reports, COVID-19 may have similar presentation in HT recipients during the early PO phase (from mild to severe forms), whether compared to HT recipients in the late PO period or to the general population.^4 - 6^

To our knowledge, this case series is the first to report results in HT recipients developing COVID-19 during the early PO period, and our experience has shown similar disease presentations compared to non-HT recipients previously reported. Larger series are required to better understand this hypothesis. It currently seems that HT should be considered for patients who cannot be discharged home in centers where durable LVAD are not available, considering individual risks and benefits, weighed for each patient and local situation.
